# Matrix Stone Occupying an Entire Renal Collecting System: A Case Report and Video of Diagnostic Ureterorenoscopy

**DOI:** 10.1155/2018/5892438

**Published:** 2018-04-23

**Authors:** Jonathan Cobley, Yih Chyn Phan, Wasim Mahmalji

**Affiliations:** Urology Department, Hereford County Hospital, Stonebow Road, Hereford HR1 2ER, UK

## Abstract

Renal matrix stones are a rare phenomenon and they present a diagnostic challenge due to their atypical radiological appearances in comparison to more commonly encountered renal tract calculi. We describe a case of known stone former presenting with loin pain and recurrent urinary tract infections who was diagnosed with a matrix stone. The video of the diagnostic flexiureterorenoscopy demonstrating the matrix stone occupying almost the entire right renal collecting system is also presented.

## 1. Case Report

A 67-year-old woman was referred to our centre for a urology opinion, having initially presented to her general practitioner with severe right-sided loin pain and recurrent* Escherichia coli *urinary tract infections. The patient had previously undergone an open right-sided pyeloplasty in 1970, followed by an open right nephrolithotomy in 1975. Past medical history consisted of depression and eczema.

Noncontrast computed tomography of the renal tract (CT KUB) revealed a solid-looking lesion in the right renal pelvis with differential diagnoses of a tumour or a poorly calcified stone. Creatinine was 61 umol/l and estimated glomerular filtration rate (eGFR) was 85 ml/min/1.73 m^2^.

**Figure 1:**
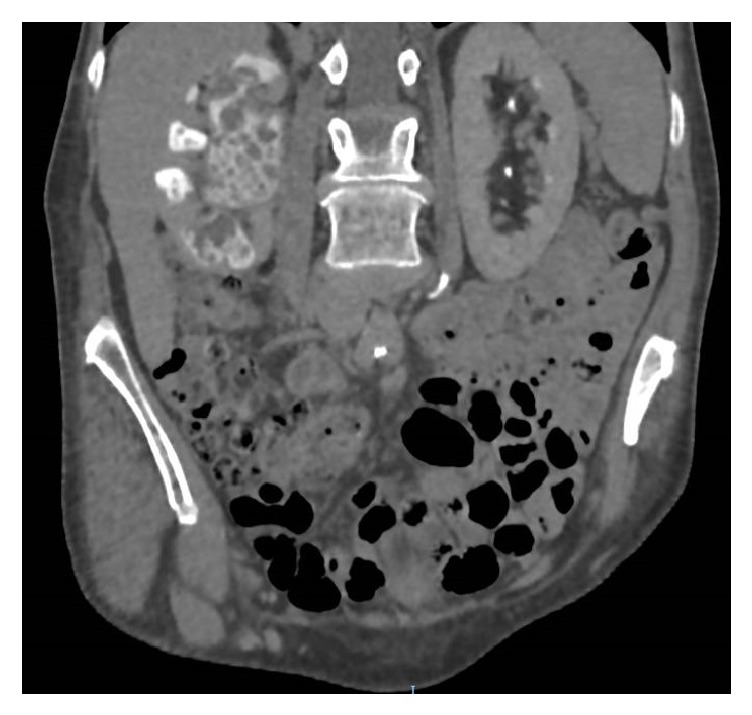
Contrast enhanced CT scan of renal tract, in coronal plane showing appearance of matrix stone throughout the right kidney.

Subsequent contrast CT urogram showed contrast outlining multiple radiolucent structures within the calyces and pelvicaliceal system, likely representing matrix stones. There was no opacification of the right ureter and although there was function in the right kidney there was extreme cortical thinning over the calyces (Figure 1).

A DMSA (dimercaptosuccinic acid) scan showed 36% function of the right kidney and a MAG-3 (mercaptoacetyltriglycine) renogram suggested moderate to severe right pelviureteric junction (PUJ) obstruction but with preserved renal function.

**Figure 2:**
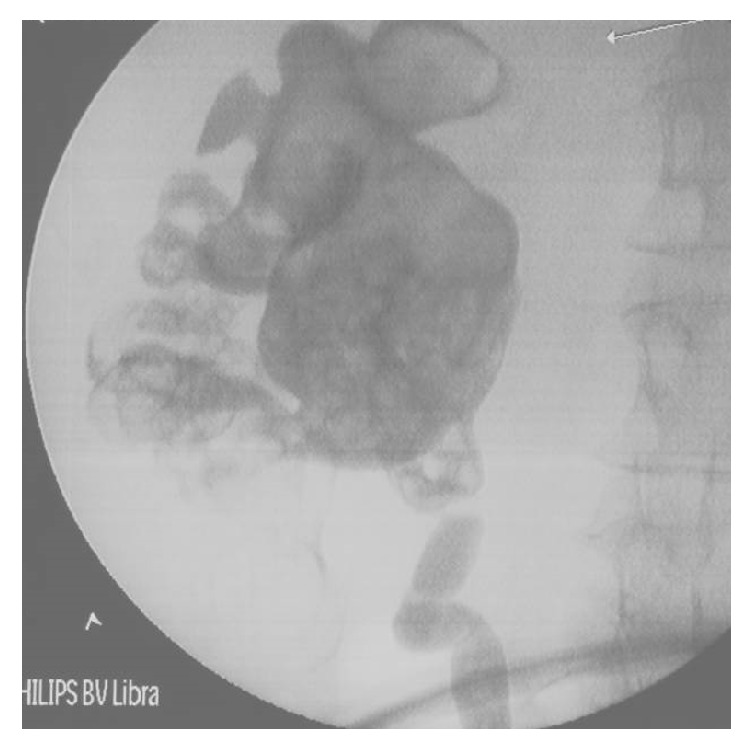
Retrograde pyelogram of right kidney with filling defects throughout. The arrow points to the site of the surgery which is the right kidney.

Following discussion at a complex case meeting, the patient underwent a right-sided retrograde pyelogram, rigid ureteroscopy, flexible ureterorenoscopy, and ureteric stent insertion. The right ureter was tortuous and dilated with a very narrow PUJ. Flexible ureterorenoscopy confirmed large volume of stone throughout the entire collecting system (see Supplementary Video (available here) and Figure 2).

**Figure 3:**
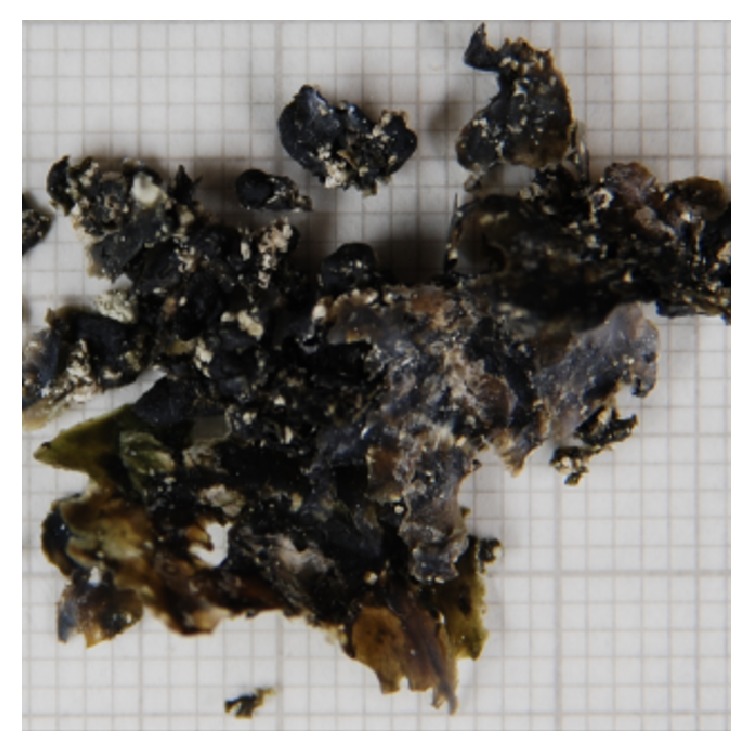
Macroscopic appearance of stone following PCNL. One small square equals 1 mm × 1 mm.

Insertion of the ureteric stent to treat her PUJ obstruction did not alleviate the patient's symptoms. The widespread extent of stone made ureteroscopic laser lithotripsy unsuitable and so the patient was managed with percutaneous nephrolithotomy (PCNL) (Figure 3).

Infrared stone analysis identified the sample to consist of mainly carbonate apatite, which is commonly formed in the presence of urinary tract infection [1].

## 2. Discussion

Renal matrix stones are a rare subtype of kidney stones which were first reported 100 years ago by Gage and Beal [2] and have also been called fibrinomas, colloid calculi, or albumin calculi. Whereas more frequently encountered calcigerous stones tend to be brittle, matrix stones by contrast are soft, amorphous, and pliable [3].

On average in this subtype of calculus, the noncrystalline, mucoprotein matrix accounts for 65% of its dry weight, compared to just 2.5% in more conventional stones [4, 5]. Analysis by Boyce and Garvey [6] found the matrix to consist of mucopolysaccharide (one-third) and protein (two-thirds). The matrix component is very similar to the matrix found in calcigerous stones and is thought to function as a foundation for the deposition of its crystalline structure or as a coprecipitant. The reason for the failure of this mechanism in matrix stones is unclear. They differ from normal stones in that they occur more frequently in females, and other risk factors include previous stone formation and surgery for stone treatment. Recurrent urinary tract infections particularly with the organisms* Proteus mirabilis* and* Escherichia coli* have also been implicated in their development [7].

Presentation is often with flank pain and/or recurrent urinary tract infections but diagnosis is challenging as the proteinaceous material which makes up matrix stones means they do not have typical radiological appearances and may be overlooked. When pure, they have been described as radiolucent; however faint calcifications have been seen on plain abdominal radiograph [8, 9]. Ultrasound shows a solid structure without the normal hyperechogenicity of stones and acoustic shadowing though imaging appearances vary depending on the mineral composition and the pattern of distribution within the collecting system [10].

Unenhanced CT may identify these calculi which are described as having a mineral rim and soft tissue centre. However, there is commonly diagnostic uncertainty, especially where differential diagnoses for filling defects include malignant tumours [11]. This was true in our case, where contrast enhanced CT with a urographic phase helped to make a diagnosis. Liu et al. [12] used magnetic resonance imaging (MRI) and found that the matrix stones showed hypointense signal in T1-weighted images, with no contrast enhancement following Gadolinium administration. T2-weighted images showed a slight hyperintense signal. Despite advances in imaging techniques there remains no specific radiological modality to definitively diagnose matrix stones and surgical intervention is frequently needed.

Retrograde ureterorenoscopy can be a useful diagnostic tool and can play a role in the clearance of smaller stones. Endourological management is advocated by a number of authors and the choice between retrograde treatment and percutaneous nephrolithotomy (PCNL) may depend on stone burden and location [8, 13, 14]. For complete clearance of larger stones, PCNL is preferred as it has been shown to achieve excellent results with low recurrence rates. Open surgery is less frequently performed whilst extracorporeal shock wave lithotripsy is ineffective [8].

## 3. Conclusion

Matrix stones are rare and are difficult to diagnose due to the lack of a single specific test; therefore a high index of suspicion is required. It is an important differential diagnosis for patients presenting with loin pain and recurrent urinary tract infections, particularly in known stone formers. Current evidence suggests that retrograde ureterorenoscopy can be used for diagnostic purposes and for clearance of smaller volumes of stone; however PCNL is the most favoured technique for patients with large stone burden.

## Supplementary Materials

Supplementary Materials Video clip from the diagnostic ureterorenoscopy demonstrating the matrix stone in the kidney.
